# Digitalization and SMEs development in the context of sustainable development: A China perspective

**DOI:** 10.1016/j.heliyon.2024.e27936

**Published:** 2024-03-11

**Authors:** Ilknur Ozturk, Omaima Alqassimi, Sana Ullah

**Affiliations:** a Faculty of Economics, Administrative and Social Sciences, Nisantasi University, Istanbul, Turkey; b College of Business Administration, University of Sharjah, Sharjah, United Arab Emirates; c Adnan Kassar School of Business, Lebanese American University, Beirut, Lebanon

**Keywords:** Digitalization, SMEs, Sustainable development

## Abstract

Worldwide economies are determined to achieve sustainable development. In this pursuit, the role of SMEs and ICT has emerged as an inevitable choice for sustainable development. The literature on the impact of SMEs and ICT on sustainable development, particularly in China's context, is scarce. Therefore, the analysis aims to investigate the impact of SMEs and ICT on sustainable development in China for the period of 1998–2020. We have applied the ARDL model for empirical analysis. The short and long-run estimates attached to SMEs are significant and positive, confirming that SMEs help to achieve sustainable development. Similarly, the estimates attached to ICT are positive and significant both in the short and long run, confirming the beneficial role of ICT in achieving sustainable development. The estimates attached to institutional quality and R&D control variables are positive and significant in the long run, but only R&D estimates are significant in the short run. In particular, digitalization and SMEs development provide a win–win situation for China to mitigate climate change in the long run and become more environmentally sustainable.

## 1 Introduction

Sustainable development represents the equilibrium between economic, social, and environmental dimensions, striving for long-term prosperity and universal well-being. It encompasses the reduction of greenhouse gas emissions, the preservation of natural resources, the promotion of social inclusivity, respect for human rights, and the establishment of sustainable infrastructure and institutions. This global endeavor aligns with the United Nations Sustainable Development Goals, a framework dedicated to eradicating poverty, safeguarding the planet, and fostering prosperity for all [1,2]. In this context, the digital transformation of SMEs is gaining remarkable prominence, signifying a pivotal nexus in the pursuit of sustainable development on a global scale. This study endeavors to unravel the intricate interplay between SMEs, digitalization, and their profound influence on sustainable development, particularly within the dynamic landscape of China.

SMEs hold immense potential to advance sustainable development by adopting eco-friendly business practices that reduce their environmental footprint, uphold principles of social responsibility, and contribute to the well-being of local communities [3]. By weaving sustainability into their operations, SMEs can sharpen their competitive edge, nurture customer loyalty, and elevate their reputation in an increasingly conscious market [4,5]. SMEs play a pivotal role in ushering society towards a sustainable future. SMEs are indispensable for employment, food production and distribution, construction, healthcare, and essential services [6]. Collectively, they wield substantial economic influence and are indispensable for driving environmental and societal transformations. In a broader context, environmentally-conscious SMEs contribute to safeguarding the climate, preserving the environment, and promoting biodiversity through their array of offerings, services, and operational approaches [7]. However, they pursue these sustainability objectives in diverse ways. Some SMEs prioritize the reduction of their production process's ecological footprint, emphasizing resource-efficient methodologies. Conversely, others concentrate on eco-friendly end-products and extend green services, such as renewable energy solutions [8].

Simultaneously, digitalization emerges as a catalytic force in optimizing resource utilization, waste reduction, and the democratization of education and healthcare access while facilitating the transition to low-carbon economies [9]. Nevertheless, it is paramount that the dividends of digitalization are distributed equitably and the ecological and societal ramifications of digital technologies are scrupulously scrutinized and counteracted. In consonance with the deliberative choice of firms to embrace digitalization, the digital perspective and environmental consciousness have ascended to critical significance in contemporary corporate strategy [10]. These cutting-edge digital technologies offer unprecedented opportunities for expanding business portfolios and enhancing growth prospects [11]. On the other hand, the eco-centric approach underscores the strategic commitment to infuse sustainability imperatives into a firm's tactical, procedural, and creative actions [12].

The rise of digitalization has diverse economic impacts, touching upon economic growth, financial development, educational outcomes, and environmental sustainability [13]. As global digitalization spreads, it offers opportunities for inclusive and sustainable development. However, these sustainable outcomes must strike a balance across the economic, social, and environmental dimensions [14]. In terms of the environment, digitalization can have both positive and negative effects. On the positive side, it can reduce CO2 emissions by lowering transaction and travel costs. Digitalization also enhances energy efficiency, smart cities, transportation systems, and industrial processes [15]. Yet, it can lead to increased energy consumption and CO2 emissions. The impact on environmental sustainability can vary, leading to discussions of an inverted U-curve relationship (Khan et al., 2020). Regarding social development, digitalization, particularly through mobile technology, has transformed lives, impacting economic well-being and human development [16]. It can promote inclusive human development, education, and even healthcare accessibility. However, the effect of digitalization on inclusive human development varies depending on various factors and dynamics. It also affects health systems by decentralizing healthcare and improving access. Policies should focus on promoting digital inclusion while safeguarding human rights, privacy, and online security (Adam and Alhassan, 2021). From an economic perspective, digitalization can foster sustainable development. It can drive economic growth, productivity, and financial development [17]. Moreover, globalization can be bolstered, further stimulating economic growth. However, without economic transformation, digitalization may have detrimental effects on economic growth [18].

In the latest literature, Luo et al. (2022) reported that digitalization enhances sustainable development in indirect ways, such as by improving the industrial structure, enhancing the degree of economic openness, and escalating the market potential, as industrial structure, economic openness, and market potential advance the impact of digital economy on sustainable development in China. He et al. [19] described that digitalization has positively enhanced the sustainable development of China's Marine Manufacturing Industry. The study reported a positive impact of digital technology on China's Marine Manufacturing Industry in which digital trade played a mediating role. Dong & Ullah [20] study proved that digitalization encourages long-run sustainable development in China. The study suggested that digitalization encourages the use of internet and IoT to decouple environmental pollution and sustainable development. Lee et al. [18] also reported the positive influence of digitalization on green growth in China. While, Li et al. (2022) demonstrated that SMEs development is positively related to the sustainable development of their host country's performance. However, technological advancement and demonstration effect play mediating roles. Zhang et al. [21] highlighted the significant role of SMEs to employment generation, regional development, and poverty reduction, aligning with the sustainable development goals. Xiang et al. [22] demonstrated the importance of environmentally friendly practices in improving the competitiveness of SMEs and contributing to overall sustainable development.

The motivation for the study lies in the persistent need to understand the dynamics between SMEs, digitalization, and sustainable development, particularly in the context of China. In recent decades, the association between enterprises, technological advancements, and sustainable development has gained significant importance, yet there exists a prominent gap in understanding the detailed implications within the SMEs and ICT sectors, particularly for a rapidly growing economy like China. The study highlights the importance of ICT and SMEs in shaping economic trajectories and their consequences for sustainable development. Previous studies have examined the impact of digitalization on environmental perspectives; there is a noticeable gap in understanding the impact of digitalization on sustainable development, especially in China. Still, there is a lack of comprehensive research on the impact SMEs development on sustainable development. Prior literature has overlooked the short-run results. This study addresses these gaps by exploring the impact of digitalization and SMEs development on China's sustainable development. The choice of the sample is placed on China as it is rapidly developing economically and technologically. China is a key carbon emitter addressing environmental problems via digitalization. Data has been collected from the World Bank, Freedom House Report, and National Bureau of Statistics of China for the period 1998 to 2020. The study employs ARDL method for empirical estimations. The main contribution of the study is that this study first assesses the impact of digitalization on sustainable development. Secondly, this provides the link between SMEs development and sustainable development. Thirdly, this study offers short and long-run results. Lastly, the findings of this study can guide policymakers in developing strategies and policies that support the digital transformation of SMEs. These policies may include investments in digital infrastructure, digital literacy programs, and regulatory frameworks to create a conducive environment for SMEs.

## 2 Theoretical framework, model, methodology, and data

SMEs make up 90 percent of all international firms and enterprises, and almost 50 percent of the global workforce is engaged in SMEs [23]. Although SMEs are critical in the development of an economy, they often have to face numerous challenges and barriers when it comes to implementing sustainable business practices and efficiently administrating natural resources. The challenges are many but not limited to scarcity of capital, shortage of technical expertise, and restricted access to markets and information [24]. The importance of Environmental, social, and governance (ESG) practices and administration of natural capital in SMEs have come under the spotlight in various academic works. These works also underscore the possible advantages of executing sustainable practices. Successful adoption of ESG practices may augment shareholders’ trust, control environmental impacts, and enhance competitiveness [25]. Achieving excellence in the administration of natural resources may help reduce waste, foster resource efficiency, and protect the ecosystem. Sustainable economic development plays a critical role in achieving long-term economic and environmental goals; nevertheless, sustainable development only becomes realistic after the effective administration of natural resources as well as promoting social justice and realizing ecological significance [26]. The incorporation of ESG practices and natural resource management are indispensable tools to attain sustainable growth, especially for SMEs. Therefore, after implementing ESG practices and efficient management of natural resources, SMEs can contribute to sustainable development.

The theoretical connection between the digital economy and sustainable development can be explained in the light of the current body of literature in the following two ways. Firstly, there is a direct connection between digitalization and sustainable development. Due to its inbuilt benefits and characteristics, digitalization introduces new sources of growth that may foster sustainable development [27]. By making use of sophisticated information technology, digitalization develop an economic structure that integrates “economies of scale, economies of scope, and the long-tail effect”. This unification boosts the balance between supply and demand in the market, resulting in high economic growth. Any development in sophisticated information technology is anticipated to enhance the mitigating efforts [28], which then help attain sustainable economic development. Secondly, there is a connection between digitalization and sustainable development via “industrial agglomerations”, which is the process of clustering industries together in certain localitieis [29]. The agglomeration helps to combine the resources from different industries, establishing robust industrial interdependencies and cooperative coalitions, resulting a rise in industrial benefits. This improves efficiency, nurtures innovation, fosters competitiveness, and encourages sustainable development. Digitalization employs data to determine user wants, facilitating the right balance between supply and demand, lowering transaction expenses for both sides and boosting demand, thereby fostering industrial agglomeration and sustainable development [30]. Thus, we follow Prashar [8] and Nchofoung & Asongu [31] who relied upon the previous standard literature and begin with the following sustainable development model:

(1) $${ANS}_{t}=\lambda_{0}+\lambda_{1}{SME}_{t}+\lambda_{2}{ICT}_{t}+\lambda_{3}{IQ}_{t}+\lambda_{4}{RD}_{t}+\varepsilon_{t}$$

where; SME, ICT, IQ, and RD represent small and medium-sized enterprises, information and communications technology, institutional quality, research and development, respectively. While $\lambda_{0}$ and $\varepsilon_{t}$ denote the intercept and the error term, respectively. $\lambda_{1}$, $\lambda_{2}$, $\lambda_{3}$, and $\lambda_{4}$ are parameters that show the impact of SME, ICT, IQ, and RD, respectively. Digitalization and entrepreneurial SMEs have also favorable effects on sustainable development. Thus, we expect an estimate of $\lambda_{1}$ and $\lambda_{2}$ to be positive. Coefficient estimates of Eq. (2) reflect the long-run effects of SMEs, ICT, IQ, and RD on ANS. Previous Eq. (1) only offers long-run estimates. Eq. (2) has been converted into the error correction format for short-run estimates.

(2) $$\Delta {ANS}_{t}=\lambda_{0}+\sum_{i=1}^{p}\beta_{1i}\Delta {ANS}_{t-i}+\sum_{i=0}^{q}\beta_{2i}\Delta {SME}_{t-i}+\sum_{i=0}^{q}\beta_{3i}\Delta {ICT}_{t-i}+\sum_{i=0}^{q}\beta_{4i}\Delta {IQ}_{t-i}+\sum_{i=0}^{q}\beta_{5i}\Delta {RD}_{t-i}+\lambda_{1}{ANS}_{t-1}+\lambda_{2}{SME}_{t-1}+\lambda_{3}{ICT}_{t-1}+\lambda_{4}{{IQ}_{2}}_{t-1}+\lambda_{5}{RD}_{t-1}+\varepsilon_{t}$$

Specification (2) is made up of two different types of coefficients, signifying its strength of producing short and long-run estimates at once. First type of coefficients are the ones adjacent to “Δ” indicator, which represent the outcomes in the short-term, while the second type of coefficients are the ones that range from $\lambda_{2}-\lambda_{5}$ normalized on $\lambda_{1}$ represent the outcomes in the long-run. There is a validity issue with the long-run results, which are considered spurious unless we prove cointegration between dependent and independent variables. The solution is provided by the Pesaran et al. [32] by developing two tests, namely the F test and ECM, to assess the validity of the findings. They also tabulated new critical values for these tests. Pre-unit testing is not a condition for this technique because it can deal with variables with diverse integrating characteristics. i.e., I(0) or I(1), which is its main strength and give it an edge over other cointegration techniques [33]. Since macroeconomic variables are either I(0) or I(1), and the results of our KPSS unit root test also align with this statement, this provides the justification for selecting ARDL over other time series techniques. Lastly, the results of this technique are valid in the case of the brief sample. This research employs diagnostic testing to check the accuracy of our outcomes. The CUSUM and CUSUM-sq are the tests for parametric stability, while the Remsey RESET and LM tests are applied to check misspecification and serial correlation, respectively. In this study, we use the ARDL methodology as the main method. However, we also use the QARDL model to enhance the reliability of our results, especially when dealing with non-normal data. The QARDL model yields precise estimations in such circumstances.

In this study, the dependent variable sustainable development is proxied by adjusted net savings as % of GNI (ANS). Entrepreneurial SMEs and digitalization are the focused variables. Entrepreneurial SMEs is measured by number of SMEs in a million, and digitalization is measured via ICT (i.e. individuals using internet in total population). The role of institutional quality and research and development are also added in regression analysis as control variables. The economic freedom index measures institutional quality (IQ), while RD is measured through research and development expenditures as a percent of GDP. The details of the variable series are reported in the Appendix (Table A1). The required data for this study is acquired from the World Bank, Freedom House Report, and National Bureau of Statistics of China for the period from 1998 to 2020. Descriptive statistics of the variables are given in Table 1. The highest mean value is recorded for IQ with a maximum score of 4.086 and a minimum of 3.932, respectively. However, lowest mean score is noted for RD with maximum range 2.401 and minimum range 0.893. Highest S.D is observed for ICT and lowest is noted for IQ.

**Table 1:** Descriptive statistics of the variables.

	Mean	Median	Max	Min	Std. Dev.	Skewness	Kurtosis	Jarque-Bera	Prob
ANS	3.057	3.076	3.339	2.652	0.181	−0.167	2.019	4.298	0.087
SME	2.358	2.426	3.778	0.556	0.915	−0.254	2.186	3.686	0.089
ICT	2.856	3.460	4.277	0.104	1.315	−0.724	2.137	11.36	0.003
IQ	3.986	3.969	4.092	3.928	0.044	0.933	2.697	14.28	0.001
RD	1.621	1.699	2.486	0.715	0.523	−0.116	1.743	6.540	0.038

## 3 Empirical results

Before estimating the regression model, it is mandatory to confirm the unit root properties of the data series. For that purpose, we applied the KPSS unit root test. The obtained results are given in Table 2. According to KPSS results, there is a mixed order of integration among variables. For instance, SME, ICT, and RD are stationary at I(0), whereas ANS and IQ are stationary at I(1). Table 3 displays the results of F-test. The obtained value for F-stat is statistically significant, confirming that long-run cointegration association exists among variables. After confirming the unit root characteristics and Cointegration results, the next step is to regress the mode through the ARDL method. The obtained results for short-run parameters are given in Table 4. It is shown that SME report a significant and positive impact on ANS, describing that a 1% increase in SME enhances ANS by 1.141% in the short-run. It confirms the positive role of SME in enhancing sustainable development. The results show that ICT significantly and positively impacts ANS, revealing that a 1% upsurge in ICT enhances ANS by 0.156%. It proves the positive contribution of digitalization in bringing sustainable development to the Chinese economy. Conversely, the IQ impact on ANS is observed as insignificant in the short run, disclosing that IQ does not play any role in bringing sustainable development in the short-run. RD and ANS are associated with positivity in the short-run. It shows that a 1% upsurge in RD tends to enhance ANS by 0.465% in the short-run.

**Table 2:** KPSS test.

	I(0)	I(1)	Critical values	
ANS	0.254	0.598	1% level	0.739
SME	0.632		5% level	0.463
ICT	0.625		10% level	0.347
IQ	0.265	0.456		
RD	0.635

**Table 3:** F bounds test.

Test-stat	Value	Signif.	I(0)	I(1)
F-statistic	6.104***	10%	2.20	3.09
K	4	5%	2.56	3.49
		2.50%	2.88	3.87
		1%	3.29	4.37

**Table 4:** Short-run estimation.

Variable	Coefficient	S.E	t-Stat	Prob.*
ANS(-1)	0.539**	0.224	2.407	0.039
SME	1.141***	0.428	2.665	0.026
SME(-1)	1.016**	0.409	2.484	0.035
ICT	0.156*	0.090	1.675	0.100
IQ	0.209	0.653	0.320	0.756
RD	0.465*	0.273	1.705	0.122
RD(-1)	0.534	0.345	1.547	0.156
RD(-2)	0.774***	0.279	2.775	0.022
C	0.119	2.752	0.043	0.966

**Note**: ***, ** and * indicate significance at 1%, 5% and 10% levels, respectively.

The output for long-run parameters is displayed in Table 5. It infers that SMEs significantly and positively influence ANS, revealing that SMEs tend to enhance sustainable development significantly in China. The results depict that a 1% upsurge in SME augments ANS by 0.613%. Hence, the long and short-run findings confirm that SME is a fundamental determinant of sustainable development. In terms of digitalization role, the results display that ICT also reports a significant and positive impact on ANS as found in the short-run. It confirms that digitalization tends to augment sustainable development in China. It is found that a 1% upsurge in ICT augments ANS by 0.433% in the long-run. The impact of IQ on ANS is found to be significantly positive in the long-run, confirming that a good quality institutional framework improves sustainable economic development. The findings imply that a 1% improvement in IQ enhances ANS by 0.205%. RD and ANS are positively associated in the long run, implying that RD significantly enhances sustainable development. It shows that a 1% upsurge in RD enhances ANS by 1.094% in the long-run.

**Table 5:** Long-run estimation.

Variable	Coefficient	S.E	t-Stat	Prob.
SME	0.613*	0.317	1.931	0.090
ICT	0.433*	0.236	1.834	0.104
IQ	0.205**	0.082	2.499	0.037
RD	1.094*	0.621	1.763	0.100

**Note**: ***, ** and * indicate significance at 1%, 5% and 10% levels, respectively.

**Fig. 1:**
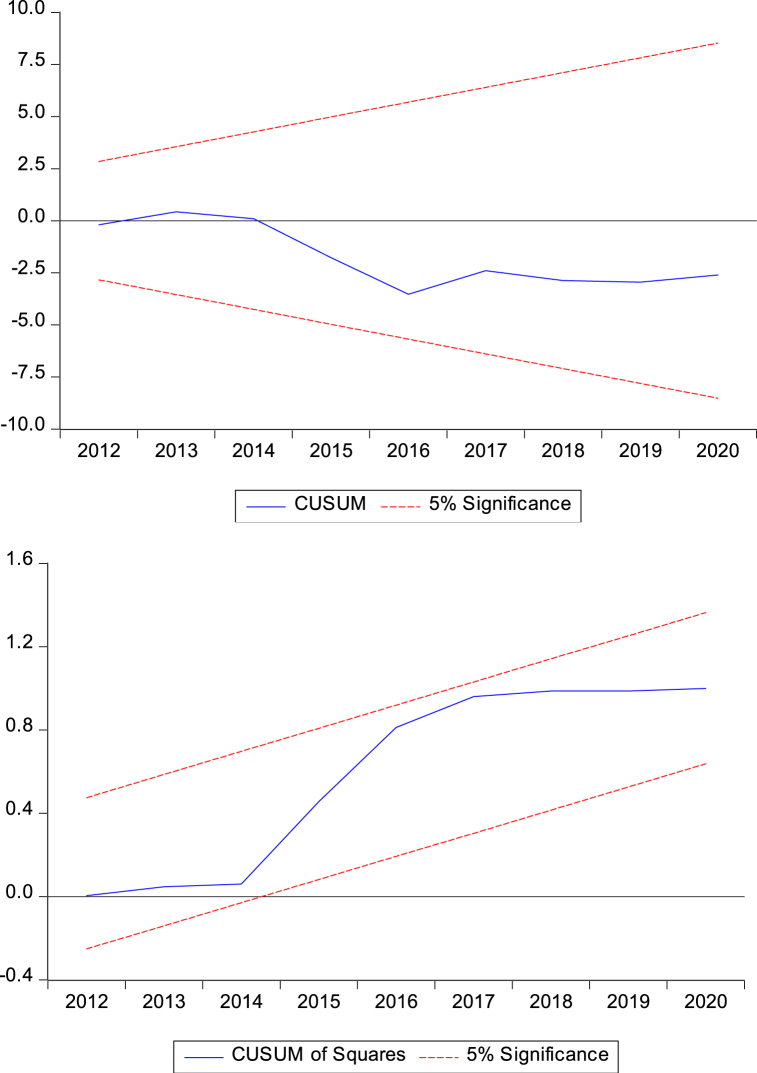
Graphs of CUSUM tests.

Table 6 displays the results of diagnostic tests such as ECM, LM, BPG, and RESET. These tests are important to confirm the validity of ARDL estimates. The ECM term is significant and negative confirming that there is the possibility of achieving stability in the long-run. The ECM term is −0.173 which shows that almost 17% stability will be achieved in the span of one year. The results of LM and BPG tests confirm that no issue of autocorrelation and heteroskedasticity is detected in the model. In the end, the RESET test result confirms that the model is correctly specified. The CUSUM tests also show stability in graphs (Fig. 1). Finally, an adjusted R2 of 0.547 shows a good fit.

**Table 6:** Diagnostic tests.

	Values	Prob.
ECM	−0.173***	0.000
LM	1.118	0.379
BPG	1.324	0.342
RESET	0.106	0.918
Adjusted R2	0.547

The output from the QARDL model is shown in Table 7. It shows that the estimates of ECM are significant and negative overall quantiles, confirming the long-term association between ANS, SME, ICT, IQ, and RD. The long-run estimates of SME are positive and significant from the 50th to 95th quantiles, while in the short-run, they are significant at all quantiles. However, the estimates of ICT are positive and significant only in the long run at all quantiles. The long-run estimates of IQ are significant from the 70th to 95th quantiles, and the estimates of RD are significantly positive at all quantiles. In the short run, the estimates of IQ are significantly positive in the last two quantiles only, while the estimates of RD are significantly positive from the 60th quantiles onwards. These results suggest that the QARDL model provides more significant results in the long run, where SME, ICT, IQ, and RD all have a favorable impact on ANS at most intensities of ANS.

**Table 7:** Long and short-run estimates-QARDL.

	Long-run estimates						Short-run estimates					
	ECM	C	SME	ICT	IQ	RD	SME	SME(-1)	ICT	IQ	RD	RD(-1)
0.05	−0.430***	6.892***	0.179	0.401***	0.855	1.295***	0.332***	0.285***	0.122	0.254	−0.656	0.424
	−3.759	6.790	1.427	12.39	1.523	10.66	5.767	4.302	1.372	1.425	−1.525	1.304
0.10	−0.263***	6.938***	0.176	0.390***	0.864	1.275***	0.332***	0.329***	0.058	0.147	−0.374	0.216
	−2.697	5.739	1.488	10.84	1.429	8.977	5.128	4.645	1.028	1.211	−1.497	0.803
0.20	−0.204*	7.155***	0.163	0.371***	0.924	1.196***	0.277***	0.266***	0.034	0.105	−0.158	0.073
	−1.954	5.132	1.387	8.509	1.310	7.286	3.832	3.503	0.499	1.036	−0.462	0.217
0.30	−0.106*	6.904***	0.047	0.398***	0.869	1.040***	0.287***	0.284***	−0.020	0.019	−0.023	0.059
	−1.714	3.854	1.259	6.981	1.293	8.545	4.986	4.712	−0.375	0.249	−0.069	0.190
0.40	−0.186*	6.996**	0.028	0.403***	0.892	1.003***	0.278***	0.283***	−0.019	0.011	0.006	0.009
	−1.678	2.422	1.033	4.375	1.195	8.172	3.286	3.430	−0.390	0.165	0.017	0.028
0.50	−0.247*	8.008**	0.116*	0.354***	1.152	1.022***	0.292***	0.298***	−0.037	−0.018	0.097	−0.034
	−1.874	2.375	1.823	3.216	1.319	7.887	4.074	4.148	−0.915	−0.315	0.263	−0.098
0.60	−0.322**	10.39**	0.237**	0.277**	1.356	1.035***	0.311***	0.335***	−0.053	−0.042	0.374*	−0.306
	−2.110	2.397	2.032	1.987	1.565	7.375	5.026	5.345	−1.279	−0.932	1.810	−1.285
0.70	−0.345***	12.32***	0.339**	0.212*	1.650**	1.032***	0.322***	0.344***	−0.059	−0.054	0.456**	−0.366
	−2.628	2.998	2.029	1.892	2.115	7.508	7.089	7.410	−1.431	−0.379	2.528	−1.437
0.80	−0.414***	13.04***	0.433***	0.157*	1.847**	1.011***	0.315***	0.338***	−0.073	0.083	0.409***	−0.289
	−2.660	2.684	3.298	1.722	1.964	6.290	7.645	7.748	−1.522	1.384	2.619	−1.544
0.90	−0.460***	16.97***	0.141***	0.230**	2.025***	0.683***	0.475***	0.396***	0.043	0.068*	0.017**	−0.009
	−2.818	4.458	3.536	2.197	3.524	2.868	3.762	4.000	0.743	1.823	2.082	−0.057
0.95	−0.512***	18.20***	0.231***	0.204**	2.331***	0.766***	0.437***	0.340***	0.131	0.241**	0.321*	0.146
	−3.317	5.826	4.356	2.415	4.682	5.003	3.011	2.622	1.210	2.427	1.957	0.761

**Note:** ***p < 0.01; **p < 0.05; *p < 0.1.

T-stats are reported below the estimates.

## 4 Results discussion

Our empirical results are backed by Prashar [8], who denoted that SMEs positively enhance sustainable development through increased economic growth, employment, and poverty reduction. Abisuga-Oyekunle et al. [6] support our findings by arguing that SMEs play a fundamental role in economic structure and generate economic and social benefits, thus augmenting sustainable development. These empirical inferences indicate that SMEs are crucial contributors to sustainable development because they create jobs, stimulate entrepreneurship, and foster innovation. By providing employment opportunities, SMEs enhance social well-being and reduce poverty. The study of Smith et al. [34] supported our results and noted that SMEs contribute significantly to technology diffusion within communities. SMEs catalyze positive environmental changes by embracing digital technologies, implementing sustainable practices, and promoting eco-friendly innovations. This aligns with SDGs 9 (industry, innovation, and infrastructure), emphasizing the importance of fostering innovation for sustainable development. SMEs are considered as an instrument of change that can boost sustainability at the national and international levels. SMEs are flexible enough and capable of adopting sustainable practices in their operations, products, and services. These empirical inferences are also supported by Westman et al. [35], who observed that a positive connection exists between SMEs and sustainability. According to Liu et al. [36], if SMEs have been provided with venture capital, they can grow in a sustainable manner due to the provision of funds, supervision, and noncapital value-added services. In addition, Platonova and Maksakova [37] suggest that full-fledged assistance for SMEs (e.g. cooperation with large enterprises), can increase their ability to overcome several hurdles and support them to participate in international value chains, fostering sustainable development. Further, the role of SMEs can not be underestimated in fostering economic growth, enhancing employment opportunities, and offering innovative ideas for sustainable development.

The study of Nchofoung & Asongu [31] supports our findings by arguing that digitalization contributes to sustainable development in many ways, such as economic growth, financial stability, and environmental sustainability. Vyas-Doorgapersad [38]. argued that ICT helps develop transportation infrastructure, smarter cities, industrial processes, electrical grids, and energy growth, thus contributing to sustainable development. Tjoa & Tjoa [39] highlight that digitalization improves the information flow and augments financial integration, thus enhancing sustainable development. Kendall & Dearden [40] denote that digitalization enhances sustainable development by improving society's social, environmental, and economic dimensions. The ICT findings also infer that ICT fosters economic growth by promoting innovation, entrepreneurship, and digital economies. E-commerce platforms, digital marketplaces, and online banking services enable businesses, especially SMEs, to expand their reach and access global markets. Digital innovation and automation enhance productivity and efficiency, driving sustainable development [15]. Moreover, ICT promotes digital inclusion by providing internet access and digital literacy training to underserved communities. Bridging the digital divide ensures that marginalized populations have access to information, education, healthcare, and economic opportunities, thus promoting sustainable development [18].

The result of institutional quality is consistent with Azam et al. [41], who noted that institutional quality favours sustainable development via effective governance. This means that good institutional quality promotes sustained economic growth and development. Sound institutions provide a favorable environment for individuals, businesses, and communities, thereby contributing to long-term sustainable development. These empirical inferences support sustainable development [42]. The findings are also backed by Niesten et al. [43], who reported the positive role of well-functioning institutions in shaping environmental sustainability practices, emphasizing that such institutions facilitate the enforcement and adoption of eco-friendly policies that lead to sustainable development. Strong institutions play a vital role in maintaining the rule of law, preserving human rights, and fighting against corruption. All these factors, according to Jahanger et al. [44], are helpful in achieving sustainable development. The results also revealed that institutional quality has a crucial role to play in achieving SDGs due to its contribution to the development of peaceful and inclusive societies, which have important characteristics of access to justice and effective, answerable, and transparent institutions. The study of Ren et al. [45] also confirmed that institutional quality is highly supportive in attracting foreign direct investment from abroad, which is crucial in bringing clean and green technology to the home nation and thus supporting sustainable development.

The result of RD is consistent with Aldieri et al. [46], who reported that the R&D role in technological advancements leads to more resource-efficient and eco-friendly practices, thus enhancing sustainable development. The study of Mo et al. [47] illustrated similar results and noted that R&D activities contribute to increased economic growth, job creation, and productivity, thus positively influencing sustainable development. These outcomes are also supported by green growth theory [48], which argues that R&D is a key pillar of sustainable development goals. According to Sánchez-Sellero & Bataineh [49], firms can achieve environmental sustainability with the help of R&D. By emphasizing R&D activities, organizations can help develop their production techniques in line with the principle of sustainability and produce products that keep pace with technological advancement, resulting in the achievement of the ecological aspect of sustainable marketing. Roper et al. [50] stated that the positive externalities that emerge as a result of R&D activities in the area of innovation diffusion have a significant role in creating new ideas by ensuring access to information, thus fostering economic growth and protecting the environment simultaneously.

## 5 Conclusion and implications

It has been shown that there is a significant correlation between global temperature variations and total emissions. Indeed, there is a strong likelihood that anthropogenic actions since the middle of the 20th century have produced GHGs, leading to an apparent rise in the world's temperature. Due to the increased burning of traditional energy sources, CO2 releases make up around 75% of the world's emissions. Over the recent 40 years, human activities have increased CO2 emissions, with non-renewable energy use accounting for up to 2/3 of the total world pollution level. In order to combat the problem of environmental degradation due to rising CO2 emissions levels, sustainable development is the most viable option for the world community. In this regard, the role of SMEs can be crucial because they are trying their best to transform their structure in the light of sustainable development. Moreover, the role of ICT is important in making economies more digitalized. Digitalization effectively converts the economy into a weightless economy, which is crucial in achieving sustainable economic development. Therefore, the analysis aims to investigate the impact of SMEs and ICT on sustainable development.

For empirical analysis, we have first conducted the KPSS unit root test that has confirmed that the series in the model is a mixture of I(0) and I(1); hence, we can apply the ARDL model, which can accommodate both I(0) and I(1) variables. The short and long-run estimates attached to SMEs are significant and positive, confirming that SMEs help to achieve sustainable development. Similarly, the estimates attached to ICT are positive and significant both in the short and long run, confirming the beneficial role of ICT in achieving sustainable development. The estimates attached to institutional quality and R&D control variables are positive and significant in the long run, but only RD estimates are significant in the short run.

We have utilized the findings of the studies to provide policy suggestions. The role of SMEs is crucial in achieving sustainable development; hence, the policymakers must facilitate the SMEs in transforming their structure to be more environmentally friendly. In this regard, SMEs must completely renovate their structures and align them with sustainable guidelines by introducing green practices. Moreover, the authorities in China must remove financial and institutional hurdles in the way of developing SMEs that pledge to perform their business activities without damaging the balance of the ecosystem, thus promoting sustainable development. Policymakers can use the findings of the study to formulate targeted policies that incentivize the digital transformation of SMEs. SMEs should align their strategies with sustainable development goals by incorporating eco-friendly practices. SMEs should make strategic decisions focusing on innovations and technology adoption that increase efficiency, reduce environmental impact, and contribute to sustainable development goals. ICT-based remedies to energy intensity, transportation, and resource usage may enhance environmental quality if used more effectively. Widespread application and acceptance of ICT in society would be an important strategy for the sustainable development of China because ICT relies more on information resources, leading to the foundation of a weightless, dematerialized, and digitalized economy. Hence, policymakers must try to increase the role of ICT in every sector of the economy that would significantly contribute to sustainable development. Implement nationwide digital literacy programs targeting SMEs owners and employees. These programs should focus on imparting essential digital skills, fostering a culture of innovation, and ensuring that SMEs can harness digital technologies effectively for sustainable development initiatives. Provide financial support, subsidies, and tax incentives to SMEs that invest in digitalization efforts aligned with sustainable practices. Establish dedicated funds to assist SMEs in adopting eco-friendly technologies, implementing energy-efficient processes, and enhancing overall digital infrastructure. Facilitate partnerships between SMEs, government agencies, and private technology firms. These partnerships can provide technical expertise, training, and access to green technologies. By implementing these policy suggestions, China can create an enabling environment for SMEs to embrace digitalization while advancing sustainable development goals. These initiatives can enhance SMEs' competitiveness, promote innovation, and contribute to a greener, more inclusive green economy.

The study has certain shortcomings which need to be highlighted. Since most macroeconomic variables move asymmetrically, our analysis does not estimate the asymmetric impact of SMEs and digitalization on sustainable development. Therefore, future studies must emphasize capturing the asymmetric relationship between SMEs, digitalization, and sustainable development. Another critical shortcoming of the study is its dependence on time series data, which only estimates the relationship in one country. The scope of the study in the context of policy implications remains limited. The study is also limited to using time-series data from 1998 to 2020. In the future, economists should try to gather data for advanced and emerging economies that would increase the effectiveness of the study's results in terms of policy implications.

## Data availability statement

Data has been collected from the World Bank, Freedom House Report, and National Bureau of Statistics of China.

## Additional information

No additional information is available for this paper.

## CRediT authorship contribution statement

**Ilknur Ozturk:** Writing – original draft, Supervision, Conceptualization. **Omaima Alqassimi:** Writing – review & editing, Visualization, Data curation. **Sana Ullah:** Software, Resources, Methodology, Formal analysis.

## Declaration of competing interest

The authors declare that they have no known competing financial interests or personal relationships that could have appeared to influence the work reported in this paper.

## Figures and Tables

**Table A1:** Description of variables

Variables	Symbol	Definitions
Sustainable development	ANS	Adjusted net savings, excluding particulate emission damage (% of GNI)
Small and medium enterprises	SMEs	Number of SMEs (millions)
ICT diffusion	ICT	Individuals using the internet (% of population)
Institutional quality	IQ	Economic freedom index
Research and development	RD	Research and development expenditure (% of GDP)

## Abbreviations

SMEs: Small and medium-sized enterprises
ICT: Information and communications technology
ARDL: Autoregressive distributed lag
CO2: Carbon dioxide
IoT: Internet of things
ESG: Environmental, social, and governance
ECM: Error correction model
KPSS: Kwiatkowski–Phillips–Schmidt–Shin
LM: Lagrange Multiplier
QARDL: Quantile autoregressive distributed lag
GDP: Gross domestic product
BPG: Breusch Pagan Godfrey
RESET: Ramsey regression equation specification error test
SDGs: Sustainable development goals
GHGs: Greenhouse gases
